# Evaluation of the therapeutic relevance of architectural aspects in child and adolescent psychiatric institutions from the perspective of architects and senior physicians

**DOI:** 10.1186/s12888-024-06282-1

**Published:** 2024-11-18

**Authors:** Dustin Fornefeld, Fabian Schmoll-Klute, Daniel Halswick, Peter Schmidt, Marie-Léne Scheiderer, Lynn Reuter, Katharina Brockmann, Anna Pfersich, Oliver Fricke

**Affiliations:** 1 Department of Special Care Dentistry, Witten/Herdecke University, Alfred-Herrhausen-Str. 44, Witten, 58448 Germany; 2 Department of Child and Adolescent Psychotherapy and Psychiatry, Gemeinschaftskrankenhaus Herdecke, Gerhard-Kienle-Weg 4, Herdecke, 58313 Germany; 3 Department of Human Medicine, Witten/Herdecke University, Faculty of Health, Alfred-Herrhausen-Str. 50, Witten, 58448 Germany; 4 Faculty of Architecture and Civil Engineering, Würzburg-Schweinfurt Technical University of Applied Sciences, Röntgenring 7, Würzburg, 97070 Germany; 5 Department of for Child and Adolescent Psychiatry and Psychotherapy, Klinikum Stuttgart, Prießnitzweg 24, Stuttgart, 70374 Germany

**Keywords:** Evidence-based design, Pediatric, Psychiatry, Hospital, Wellbeing, Building

## Abstract

**Objective:**

The concept of “Healing Architecture” addresses the relevance of design and architectural issues on the outcome of medical and therapeutic treatment in hospitals. The questionnaire ARCHI was developed to record data on the opinion of different groups of users on the architectural design of their therapeutic environment in departments of child and adolescent psychiatry.

**Method:**

A Questionnaire-based cross-sectional study was conducted in two phases between 2020 and 2022 using ARCHI to gather the perspectives of senior physicians and architects on the significance of architectural design in German child and adolescent psychiatric facilities.

**Results:**

In the survey of the senior physicians, 49 heads of child and adolescents psychiatric departments initiated the survey, and 73.5% (*n* = 36) of them completed the questionnaire in its entirety. During the survey for the architect-specific, 145 individuals commenced the survey, and 60.7% of them completed the questionnaire (*n* = 88). Significant differences between architects and senior physicians became visible for four of the 93 items, namely “environment of the hospital”, “structure of building”, “labelling of floors” and “visible cleanliness”.

**Conclusions:**

Although senior physicians and architects were characterized by the same opinions on the relevance of architectural design on therapeutic issues for the major part of issues, differences could be obtained for several aspects which are important for the architectural planning of new departments and hospitals. It remains open why both professional groups had different point of views on these four aspects of hospital design which should be clarified to improve the process of hospital development in the future.

## Introduction

Under the term “Healing Architecture”, theoretical approaches, scientific foundations, and best-practice examples increasingly converge, focusing on the impact of architecture and various building components on the physical and psychological well-being of individuals. The scholarly exploration of this subject began with the 2009 work “Helende arkitektur” by Frandsen et al. [1], which initially presented a comprehensive foundation of concepts and methods from the field of Healing Architecture. Today, architecture is considered a non-medical measure that nevertheless influences medical outcomes [2]. Within the context of “Healing Architecture” and “Evidence-Based Design”, this influence on the healing process is illuminated through specific architectural features and interior design elements [2]. This encompasses analysis, specifically involving an examination of the current building structure on users (in this case, patients), as well as prospective planning and the derivation of “decisions for later clinic construction […] from evidence regarding potential effects of architecture on people’s health” (Translation: DF) [3].

In the field of “Healing Architecture”, factors influencing both fundamental building structures, such as the individual layout of spaces and adjacent communal areas or air conditioning [4, 5], as well as internal influences, such as patient-driven spatial design (e. g., through plants, pictures, books and the possibility to ensure privacy and intimacy) and the arrangement of furniture, enabling autonomous regulation of proximity, distance and noise reduction [4–10], and external influences and the environment, such as the presence of a natural environment in the form of forests, rivers and lakes, and the ability to flexibly utilize daylight and the surroundings [5, 11–15], are examined. A transcending effective element for the therapeutic process is that users already feel invited and welcomed through the fundamental architecture [5].

Using the example of German child and adolescent psychiatry, the importance of addressing architectural conditions in psychiatric facilities becomes evident. In 2022, a total of 60,711 patients were treated in inpatient basis in German child and adolescent psychiatry, representing a 67% increase compared to 2004 [16]. A total of 2,041,972 treatment days were recorded, corresponding to an average length of stay of 31.9 days [16]. It is therefore evident that children and adolescents, in comparison to other inpatient treatments, undergo full inpatient treatment for an exceptionally long duration (+ 443% compared to the average inpatient stay in 2022). Child and adolescent psychiatric facilities often feature smaller ward units compared to adult psychiatry and are age-group-specifically managed [17]. Due to the comparatively long length of stay and the pedagogical-psychotherapeutic organized, partially family-analogous daily programs and routines, it is evident that child and adolescent psychiatry temporarily becomes a home away from home. In such temporary residences, the architectural design places a stronger emphasis on the layout of the existing building infrastructure than in facilities with shorter lengths of stay.

Nevertheless, the architectural influence of inpatient therapy for children and adolescents seems to remain a niche topic. Often, architectural aspects are only considered in the context of suicide prevention [18]. In these complex planning and construction processes, different professions apply various standards and criteria to the construction or renovation of child and adolescent psychiatric facilities. This often involves individual concepts of architecture and beauty, with the result being understood more as an artwork that reflects the outcome of a subjective engagement of architects with aesthetics and the history of architecture [19].

In this discussion, Vollmer et al. [3] point out a potentially known but highly significant circumstance, namely that “*architects are not primarily trained to interpret scientifically determined environmental factors in a user-specific manner and to implement them in their designs according to user needs*” while, on the other hand, “*clinic staff, often tasked with the initial steps of new construction or renovation planning, [are not trained] to think spatially and formulate design criteria*” (Translation: DF). Vollmer et al. [3] highlight the discrepancy between the architect-specific planning perspective and the user-specific perspective. This study aims to scrutinize this circumstance and attempt to determine whether, and if so, in which areas there is a discrepancy between these two perspectives.

## Material and methods

The present questionnaire-based, cross-sectional study was conducted in two survey phases. In 2020/2021, the senior physicians of German child and adolescent psychiatric facilities were initially contacted. In the context of surveying senior physicians, the entire collective of all German child and adolescent psychiatric institutions was consulted, according to the listing of the German Society for Pediatric and Adolescent Medicine [Deutsche Gesellschaft für Kinder- und Jugendmedizin e. V.], as of September 26, 2020. At the time of the survey, 547 child and adolescent psychiatric facilities were identified, of which 350 child and adolescent psychiatric institutions remained after excluding closed or outpatient facilities. Subsequently, all 350 institutions were individually contacted, and the senior physicians were invited to participate. In the cover letter, participants were informed about the survey’s topic, the implementation, and the scientific relevance of the research theme.

In the second survey phase (2022), the architect-specific survey was implemented in collaboration with all regional Chambers of Architects in Germany, Architects for Hospital Construction and Healthcare [Architekten für Krankenhausbau und Gesundheitswesen e. V.] and other regional associations of German Architects such as the Association of German Architects [Bund Deutscher Architekten e. V.] and the Architects and engineer association [Architekten- und Ingenieurverein Hamburg e. V.]. The willingness of the aforementioned associations to support the present study enabled us to approach a comparatively large cohort of the desired target group. In the spirit of participation, the associations were informed in advance about basis parameters such as type, implementation and scope. At no time did the associations exert influence on the design, analysis, and interpretation of the data.

### Survey using an online questionnaire

As part of the survey of senior physicians, all senior physicians of the 350 selected German child and adolescent psychiatric institutions were manually invited to participate in the survey. The initial correspondence was sent via email by the author team on November 17, 2020. The survey took place between November 17, 2020, and February 01, 2021. A participation reminder was sent on January 07, 2021. As part of the architect-specific survey, respective associations were requested to distribute the invitation, information, and survey link through their internal email distribution lists. The survey was conducted between September 19, 2022, and December 05, 2022. Three reminders were sent out after 10 days, 21 days, and six weeks.

Both surveys were conducted online using the SoSci platform (SoSci Survey GmbH, Munich). All data were collected in accordance with the General Data Protection Regulation (GDPR). The ethics committee of Witten/Herdecke University exempted our survey from the basic authorisation requirement of an ethics committee, as we only conducted a anonymous survey of experts of legal age and competence and there were no professional concerns in this regard. Such an exemption appears to be common and has also been granted in other surveys with adult and competent experts, as there were no legal objections here either (cf. [20, 21]). All Participants were informed in advance that participation was voluntary and anonymous. Additionally, they had to inform that they were of legal age and had read, understood and accepted the consent form. Due to data protection concerns, we opted against collecting sociodemographic information. Given the limited and circumscribed sample in the survey of senior physicians, from which drawing inferences about specific individuals based on sociodemographic data could potentially occur, we refrained from such data collection. We maintained this approach for the architect-specific survey to ensure comparability.

The previously validated questionnaire for evaluating the therapeutic relevance of architectural aspects in clinics or care facilities for child and adolescent psychiatry, psychosomatics, and psychotherapy for professionals working therapeutically within in the System – ARCHI [Fragebogen zur Evaluation der therapeutischen Relevanz von architektonischen Aspekten in Kliniken bzw. Versorgungseinrichtungen für Kinder- und Jugendpsychiatrie, Psychosomatik und Psychotherapie für im System therapeutisch arbeitende Personen] was employed [19]. The questionnaire consisted of 27 questions, including 22 closed-ended and five open-ended questions. In this process, multiple aspects are addressed simultaneously within a single question, resulting in a total of 93 aspects being examined across the 22 closed-ended questions (see Table 1 for an example question translated from German). In 17 questions, the relevance of architectural aspects for different functions could be ranked on a three-point Likert scale as “not relevant”, “relevant” or “fully relevant”. In the remaining five open-ended questions, respondents were asked to assess the priority of the surveyed aspects, ranking them according to their importance and impact on therapeutic treatment. The survey conducted using the ARCHI questionnaire is considered reliable (93 items; ⍺ = .849). Participation in the survey took approximately 20–30 min and was conducted in German. In the analysis of our study, the authors opted to incorporate all questionnaires completed until the end and not exclusively those that were entirely filled out, resulting in slightly fluctuating sample sizes. Nevertheless, the authors maintained transparency in this regard, providing the corresponding sample size for each item.

**Table 1:** Example of the ARCHI questionnaire

Question 12: Which of the following aspects in decoration and interior design are relevant to you from a therapeutic perspective?			
	Not relevant	Relevant	Fully relevant
Flowers and Plants			
Patient-created decorations in therapeutic settings			
Visible cleanliness			
Nature motifs as wall decorations			
Question 13: Which of the following aspects can help patients navigate both within the ward and throughout the building?			
Floor labelling			
Signage in general			
Symbols & pictograms			
Colour coding			

### Data analysis

To ensure data security, the datasets were stored on a separate storage medium with restricted access limited to specific individuals from the group of authors. The collected data were statistically processed using SPSS Version 29 (IBM Corporation, New York, NY, USA). Descriptive analyses were conducted, such as frequency, skewness, and mean. Cross-sectional correlations were also computed. The non-parametric Mann–Whitney-U-Test was employed to examine significant differences in central tendencies between the data of different samples. Given that the sample size was sufficiently large (n1 + n2 > 30), significance was assessed by z-standardizing the U value. The z-value was then compared to the critical value of the z-distribution for significance testing. For the chosen two-tailed significance level of α = 0.05, the critical value was 1.96. To test the difference in responses among different dependent samples with respect to a single professional group, the sign test was performed. To avoid a type 1 error at the chosen alpha level of 5%, the conservative Bonferroni correction was selected and applied.

## Results

In the survey of the senior physicians of German child and adolescent psychiatric facilities, a total of 55 calls were documented. Of these, 49 individuals initiated the survey, and 73.5% (*n* = 36) of them completed the questionnaire in its entirety. During the survey period for the architect-specific inquiry, a total of 328 calls were documented. Of these, 145 individuals commenced the survey, and 60.7% of them completed the questionnaire (*n* = 88). On the part of the senior physicians, 14.0% of the contacted senior physicians initiated the survey, with 10.3% completing the survey in its entirety. A response rate in the case of the survey of the architects cannot be specified for this survey since various associations were involved, and participants may have been members of multiple aforementioned associations. In the overall analysis, considering the existence of optional questions, a total sample size of 124 individuals emerged, comprising 36 senior physicians and 88 architects.

The selection of analysis variables was based on a meticulous evaluation of all available survey data. The objective was to identify variables that exhibited significant differences between the groups. Following a comprehensive review of the data, 14 out of 93 items were selected for their particularly distinct difference in response distributions between the groups. These questions were identified as meaningful identicators for relevant thematic areas and formed the basis for further statistical analysis. Initially, ten out of the 14 selected items exhibited a significant difference (s. Tables 2 and 3). After applying the Bonferroni correction, a significant difference remained in four out of the 93 items, which will be subject to further investigation ("rural environment", "division into pavillions", "visible cleanliness", "floor labeling").

**Table 2:** Listing of the 14 selected Items with their corresponding significance levels

Category (Item)	Mann–Whitney-U-Test (u—value)	Mann–Whitney-U-Test (z—value)	two-tailed significance level (α = 0.05)	P_raw_ x Number of tests (14) = P_corrected_	Bonferroni-corrected α-level (0.05/14 = 0.00357)
Digital Equipment	986.00	−3.55	< 0.001	0.005	Not significant
Quality of therapeutic work	1383.00	-.95	0.343	4.809	Not significant
Security and Protection	1266.00	−2.89	0.004	0.005	Not significant
Rural Environment	853.50	−4.30	< 0.001	< 0.001	significant
Division into pavillions	480.50	−6.20	< 0.001	< 0.001	significant
Block with courtyard	1027.00	−2.85	0.004	0.062	Not significant
Accident prevention	1371.50	-.43	0.669	9.366	Not significant
Child-friendly room design	1336.00	-.85	0.393	5.505	Not significant
Separate lounge areas	1320.00	−1.29	0.196	2.748	Not significant
Visible cleanliness	932.50	−3.81	< 0.001	0.002	significant
Floor labelling	605.50	−5.53	< 0.001	< 0.001	significant
Suicide prevention	1016.00	−3.04	0.002	0.033	Not significant
Organic materials	967.00	−3.65	< 0.001	0.004	Not significant
Space for movement	1062.00	−3.29	0.001	0.017	Not significant

**Table 3:** Ordinally scaled items that exhibited differences in the graphic-descriptive analysis

Item	Professional group	Not relevant	relevant	Fully relevant
Question 1: *What relevance do the following factors have for the quality of the therapeutic process in child and adolescent psychiatric treatment?* Item: Digital equipment	Architects (*n* = 83)	13 (15.7%)	58 (69.9%)	12 (14.5%)
	Senior physicians (*n* = 36)	0 (0.0%)	22 (61.1%)	14 (38.9%)
Question 2: *What factors in child and adolescent psychiatric treatment does architecture have a significant impact on?* Item: Quality of the therapeutic work of the staff	Architects (*n* = 85)	7 (8.2%)	44 (51.8%)	34 (40.0%)
	Senior physicians (*n* = 36)	2 (5.6%)	24 (66.7%)	10 (27.8%)
Question 2: *What factors in child and adolescent psychiatric treatment does architecture have a significant impact on?* Item: Security and Protection	Architects (*n* = 85)	1 (1.2%)	3 (3.5%)	81 (95.3%)
	Senior physicians (*n* = 36)	0 (0.0%)	8 (22.2%)	28 (77.8%)
Question 3: *What relevance should the following factors have for the choice of a clinic location?* Item: Rural Environment	Architects (*n* = 85)	11 (12.9%)	46 (54.1%)	28 (32.9%)
	Senior physicians (*n* = 36)	13 (36.1%)	23 (63.9%)	0 (0.0%)
Question 5: *What therapeutic benefits do you attribute to the following external architectural concepts of the clinic’s building structure?* – Item: Division of the rooms into separate pavilions	Architects (*n* = 82)	9 (11.0%)	29 (35.4%)	44 (53.7%)
	Senior physicians (*n* = 36)	25 (69.4%)	8 (22.2%)	3 (8.3%)
Question 5: *What therapeutic benefits do you attribute to the following external architectural concepts of the clinic’s building structure?* – Item: Block with courtyard/ atrium	Architects (*n* = 82)	9 (11.0%)	31 (37.8%)	42 (51.2%)
	Senior physicians (*n* = 36)	11 (30.6%)	15 (41.7%)	10 (27.8%)
Question 6a: *What relevance do you ascribe to the following aspects of a child psychiatric ward? –* Item: Accident prevention	Architects (*n* = 87)	2 (2.3%)	42 (48.3%)	43 (49.4%)
	Senior physicians (*n* = 36)	1 (2.8%)	6 (16.7%)	29 (80.6%)
Question 6b: *What relevance do you ascribe to the following aspects of a child psychiatric ward? –* Item: Child-friendly room design	Architects (*n* = 87)	0 (0.0%)	15 (17.2%)	72 (82.8%)
	Senior physicians (*n* = 36)	1 (2.8%)	3 (8.3%)	32 (88.9%)
Question 8: *What relevance do the following architectural possibilities have in involving the family in therapy?* Item: Separate lounge areas for parents	Architects (*n* = 85)	20 (23.5%)	35 (41.2%)	30 (35.3%)
	Senior physicians (*n* = 36)	7 (19.4%)	24 (66.7%)	5 (13.9%)
Question 12: *Which of the following aspects in decoration and interior design are relevant to you from a therapeutic perspective?* Item: Visible cleanliness	Architects (*n* = 84)	3 (3.6%)	44 (52.4%)	37 (44.0%)
	Senior physicians (*n* = 36)	1 (2.8%)	5 (13.9%)	30 (83.3%)
Question 13: *Which of the following aspects can help patients navigate both within the ward and throughout the building?* Item: Floor labelling	Architects (*n* = 84)	13 (15.5%)	35 (41.7%)	36 (42.9%)
	Senior physicians (*n* = 36)	22 (61.1%)	13 (36.1%)	1 (2.8%)
Question 14: *How relevant are the following reasons that advocate for accommodation in multi-bed rooms?* Item: Suicide prevention	Architects (*n* = 83)	6 (7.2%)	44 (53.0%)	33 (39.3%)
	Senior physicians (*n* = 36)	12 (33.3%)	16 (44.4%)	8 (22.2%)
Question 16: *What significance do you attribute to the following materials for ward design?* Item: Organic materials	Architects (*n* = 85)	3 (3.5%)	25 (29.4%)	57 (67.1%)
	Senior physicians (*n* = 36)	3 (8.3%)	22 (61.1%)	11 (30.6%)
Question 17: *What relevance do the following factors in the design of the outdoor area have?* Item: Space for movement / Physical activity Area	Architects (*n* = 85)	4 (4.7%)	32 (37.6%)	49 (57.6%)
	Senior physicians (*n* = 36)	1 (2.8%)	3 (8.3%)	32 (88.9%)

### Relevance of various factors in the selection of the clinic location

Question 3 of the ARCHI questionnaire examines the relevance of various factors in the selection of a clinic location. Four distinct factors are investigated in this context: connectivity to other clinical facilities, urban environment, rural environment, and accessibility via public transportation. The sample consisted of 36 senior physicians and between 84 and 88 architects, resulting in a total sample of 120–124 participants ("connectivity to other clinical facilities" *n* = 120; "rural environment" *n* = 121; "connectivity to public transportation" and "urban environment" *n* = 122).

For three of the four factors surveyed, there are no significant inter-group-differences, suggesting that both professional groups share a similar stance regarding connectivity to other clinical facilities, an urban environment and public transportation accessibility. More than two-thirds of respondents (82.5%; *n* = 99) would assert that connectivity to other clinical facilities is either “relevant” or “fully relevant”. Almost all respondents agree that connectivity to public transportation is “relevant” or “fully relevant” (95.1%; *n* = 116). Approximately half of the respondents (45.9%; *n* = 56) indicated that an urban environment should have no relevance in the choice of the clinic location.

**Fig. 1:**
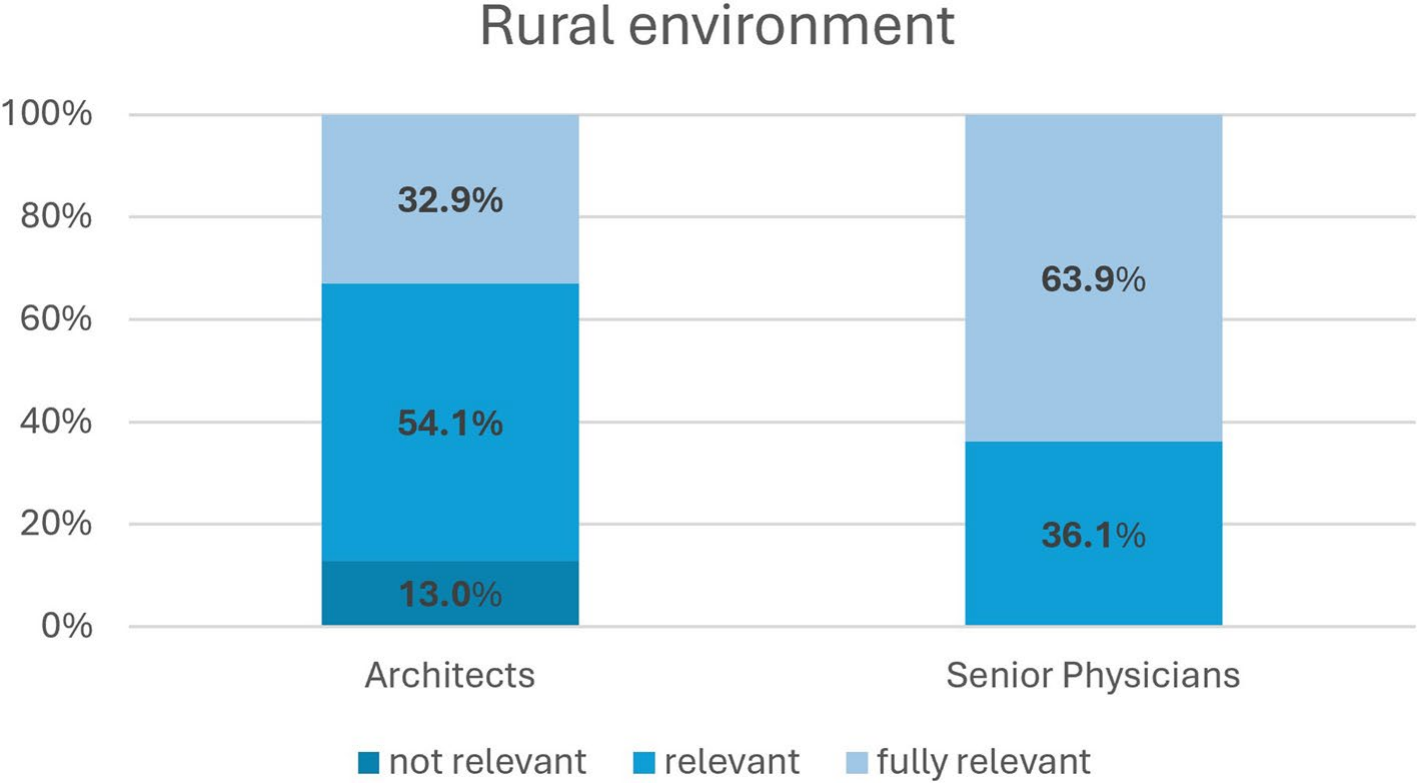
Presentation of the distribution of the Likert scale for Question 3 (Rural environment)

Regarding the significance of a natural and rural environment in the selection of the clinic location, there are divergent views between the two professional groups. Almost one-third of participating architects (32.9%; *n* = 28) would consider a rural environment to be “fully relevant” in the choice of hospital location, whereas none of the senior physicians rated a rural environment as “fully relevant” (s. Fig. 1). Conversely, over one-third of participating senior physicians (36.1%; *n* = 13) would say that the rural environment is “not relevant” in selecting a hospital, while only 12.9% of architects (*n* = 11) would express this view. In the quantitative analysis, the responses of the two professional groups were found to differ significantly (*p* < .001).

### Therapeutic benefits of different external architectural concepts in building structure

The ARCHI questionnaire assesses perspectives on the therapeutic benefits associated with various external building forms. The following external building forms are considered: "division of spaces into separate pavilions", "L-shape", "U-shape", "oval or elliptical shape", "E-shape", "star shape" and "atrium". The underlying dataset consisted of 36 responses from senior physicians and between 81 and 84 responses from architects, resulting in a total sample size ranging between 117 and 120 for different items ("atrium" and "L-shape" *n* = 120; "oval shape" *n* = 119; "star shape" and "E-shape" *n* = 118; "U-shape" *n* = 117).

**Fig. 2:**
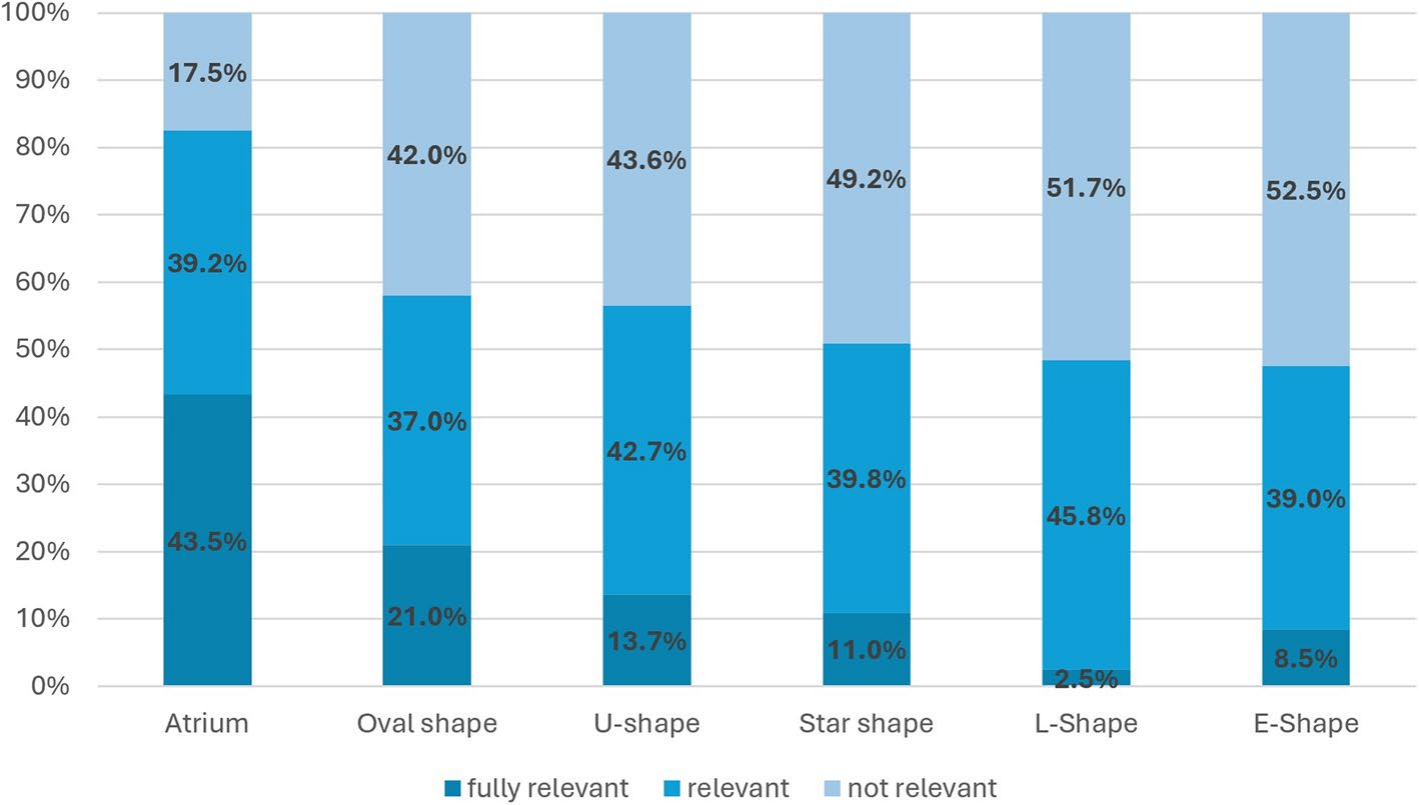
What therapeutic benefits do you attribute to the following external architectural concepts of the clinic’s building structure? Listed according to the highest therapeutic relevance (Atrium *n* = 120; oval shape *n* = 119; U-shape *n* = 117; starshape *n* = 118; L-shape *n* = 120; E-Shape *n* = 118)

Regarding the assessment of therapeutic relevance for building forms such as "atrium", "oval shape", "U-shape", "star shape", "L-shape" and "E-shape" no significant differences between professional groups were observed after applying the Bonferroni correction (see Table 2). The surveyed senior physicians and architects attribute the highest therapeutic benefit (82.5%) to the building form “atrium” and the lowest (47.5%) to the building form “E-shape” (see Fig. 2). In the evaluation of the therapeutic benefits of dividing spaces into separate pavilions, a significant difference between the two groups emerges.

A noteworthy majority of participating architects (53.7%; *n* = 44) assert that they perceive a “fully relevant” therapeutic benefit in a building structure with smaller units, such as pavilions, in contrast to a mere 8.3% (*n* = 3) of senior physicians who share this perspective. Over two-thirds of participating senior physicians (69.4%; *n* = 25) attribute no significant therapeutic benefit to the division of spaces into separate pavilions. The quantitative analysis reveals a noteworthy disparity in responses between the two professional cohorts, reaching statistical significance (*p* < .001).

### Therapeutic relevance of decoration and interior design

Regarding the relevance of various aspects in decoration and interior design, the ARCHI questionnaire examined four distinct factors: "flowers and plants", "visible cleanliness", "nature motifs as wall decoration", and "decoration created by patients in therapeutic everyday life". To address the research question, a total of 121 responses (36 senior physicians, 85 architects) were collected for the factors of "flowers and plants", "nature motifs as wall decoration", and "decoration created by patients in therapeutic everyday life", while 120 responses (36 senior physicians, 84 architects) were obtained for the factor of "visible cleanliness".

Concerning the first three factors mentioned, respondents are in agreement: Participants attribute the highest therapeutic relevance to the incorporation of decoration created by patients in therapeutic everyday life (90.9% fully relevant or relevant). Furthermore, more than four-fifths of respondents also perceive therapeutic in the factor of flower and plants (88.4% fully relevant or relevant). However, only slightly more than half of the respondents (55.4% fully relevant or relevant) ascribe therapeutic relevance to utilizing nature motifs as wall decoration in interior design. Regarding the factor of “visible cleanliness”, a significant difference between the surveyed groups is evident (see Table 2). In this context, more than four-fifths of all participating senior physicians (83.3%) would assert that visible cleanliness is “fully relevant”. Among architects, the therapeutic relevance is distributed as follows: 44.0% consider it “fully relevant”, 52.4% deem it”relevant”, and 3.6% deem it “not relevant”. In the quantitative analysis, a significant disparity in responses between the two professional groups is evident (*p* < .001).

### Orientation in the building and on the ward

Different systems can be employed for orientation within a medical facility, both on the ward and throughout the building. ARCHI introduces four distinct aspects for evaluation, providing respondents the opportunity to assess them: "floor markings", "general signage", "symbols and pictograms", and "colour coding". Data for the evaluation of "symbols and pictograms" and "colour coding" are available from 36 senior physicians and 85 architects (*n* = 121), "floor markings" from 36 senior physicians and 84 architects (*n* = 120) and "general signage" data from 36 senior physicians and 83 architects (*n* = 119).

Regarding "general signage", "symbols and pictograms", and "colour coding", there are no inter-group-differences, suggesting substantial agreement between the groups. Concerning the various means of orientation on the ward and in the building, respondents seem to perceive minimal differences, with the three aforementioned options appearing to be equally valued. However, in terms of providing orientation through floor markings (e.g., arrows), the two surveyed groups exhibit differences in their perspective.

In this regard, three-fifths of all senior physicians (61.1%; *n* = 22) stated that this was “not relevant”, contrasting with only 15.0% among architects (*n* = 13) who expressed the same view. The majority of architects (42.9%) find specific floor markings to be “fully relevant”. In contrast, only 2.8% of the senior physicians (*n* = 1) hold a similar perspective. The quantitative analysis reveals a significant disparity in responses between the two professional groups (*p* < .001).

## Discussion

The present study aligns with the recommendation put forth by Fricke et al. [5], emphasizing the significance of research in the realm of Healing Architecture. In doing so, this study addresses the disparity posited by Vollmer et al. [3] between those involved in planning and those utilizing successful hospital architecture. The comparative analysis of two substantial samples, comprising senior physicians and architects, facilitated a more in-depth exploration of this disparity, contributing to the scientific underpinning of successful hospital architecture.

In pursuit of this objective, we adhered to the ARCHI questionnaire. This questionnaire allowed the comparison of 93 factors associated with successful hospital architecture. Following the initial analysis, 14 items exhibited an significant difference, and after applying the Bonferroni correction, four items remained, indicating no significant difference across 89 items in total. In an broad overview, one might cautiously assert that the presumed disparity between planners and user appears to be less pronounced than initially anticipated. Subsequently, a brief discussion will delve into the most significant differences, including initial hypotheses regarding their genesis.

### Rural environment

A significant difference exists in the assessment of relevance regarding the choice of the clinic location. Participating architects perceive the relevance of a rural environment higher than participating senior physicians (*p* < 0.05). An (undeniable) factor in this context is certainly that the availability of rural properties, large enough to enable innovative and appealing hospital architecture, is greater than in urban areas. From an architectural perspective, it becomes evident, as research over the past decades has predominantly confirmed, that a clinic located in a rural setting, due to its connection with nature and the presence of a green environment is fundamentally more aesthetically pleasing. It facilitates a “retreat from the city” and, thereby, stress reduction, promising positive effects on (mental) health [11–14].

On the side of senior physicians, in the juxtaposition of advantages and disadvantages, the opportunity for coordination with other medical facilities and the concentration of (medical, personnel, technical) resources, which is more readily available in urban areas, seems to outweigh the appeal of a rural setting. Cautiously stated, one could hypothesize at this juncture that architects are fundamentally informed about the positive effects of a nature-centric environment (by research, education or training), a knowledge gap that exists among medical professionals. This leads to the underestimation by senior physicians of the influence and importance of a nature-centric environment. Other Hypotheses could be, that Clinics located in rural areas likely face challenges with connectivity, which can impede access. This issue is particularly pertinent in child and adolescent psychiatry, where the regular involvement of the family system is essential. The geographical isolation of a rural clinic may thus represent a significant disadvantage. Another Hypotheses could be seen from a historical Perception: Historically, senior physicians may perceive a rural environment as indicative of a tendence to segregate and exclude patients from the broader community. This perception could influence the evaluation and choice of clinic locations. A last hypothesis would be, that senior physicians have safeguarding concerns. From a safeguarding perspective, an isolated location might be associated with a higher risk of abuse. The lack of interaction with an non-clinical environment can reduce oversight and increase vulnerability to harmful situations.

### Building structure

Concerning the building structure, it becomes evident that architects assess the therapeutic relevance of pavilions significantly higher than senior physicians, who tend to favour larger structures and nearby units (*p* < 0.05). It could be hypothesized that senior physicians may prefer patient concentration due to the potential for (cost-)efficiency. Conversely, architects might prioritize aspects of personality and privacy, facilitating adjacent professions (e.g., nursing) to respond more individually to specific needs.

The latter aligns well with the current scientific investigations. Scientific studies indicate that smaller ward units, which convey a home-like atmosphere and are specifically tailored to psychiatric conditions (not a mere repurposing of an existing somatic ward), in adult psychiatry lead to more social interaction among patients and staff and patients express greater satisfaction with the local conditions [22, 23]. An exemplary study illustrates how a large adult psychiatric ward was subdivided into smaller units through the rearrangement of furniture, social spaces and spatial partitions, resulting in increased social interaction, with patients spending more time in communal areas [24]. Thus, the architectural perspective aligns with current research findings in the field of Healing Architecture at this juncture.

### Visible cleanliness

Regarding visible cleanliness, the surveyed senior physicians attribute higher therapeutic relevance to interior design and decoration compared to the surveyed architects (*p* < 0.05). From a medical standpoint, this appears comprehensible as cleanliness plays a crucial role in the prevention and control of infections. Moreover, visible cleanliness can contribute to enhancing trust in medical care. The dissonance between the perspective of architects and senior physicians at this juncture vividly underscores the incongruity between aesthetically user-specific design aspirations (architects) and the desire for materials that are both aesthetically pleasing and easy to clean and sterilize (senior physicians).

### Floor markings

Another examined aspect delves into possibilities for patients to navigate the building and ward more easily. According to the surveyed senior physicians, the use of easily understandable floor markings (e. g., arrows) to assist patients in navigating the building is a somewhat negligible approach. In their view, alternative measures, such a guidance from reception or other staff, are preferably relied upon to help patients navigate the premises. On the architects’ side, who attribute significantly more relevance to the option of providing orientation through arrows, the focus likely revolves around aspects of inclusion and accessibility (*p* < 0.05). In this regard, they adhere to the principle: Complex structures necessitate clear signage. Navigational aid in hospitals and its impact on humans has been investigated in several studies. It could be shown that wayfinding information can increase the well-being of patients and their relatives as well as reduce their level of stress. Regarding the type of wayfinding systems a combination of signage and the option of asking staff for help was recommended by the scientists [25].

## Future research

The examination of hospital architecture’s nuanced effect remains a nascent endeavour, lacking a robust scientific foundation for practical implementation. Noteworthy is the prevalence of methodological deficiencies in Healing Architecture studies, often constrained to retrospective pre-post surveys, with a scarcity of randomized controlled trials, diminishing their interpretive capacity [13, 15]. To bolster the scientific underpinning, rigorous studies, such as longitudinal or randomized controlled trials, are imperative. Cross-cultural comparisons are necessary for validating potential cultural disparities. Explore concrete implementation strategies for effective hospital architecture and practical approaches to ward renovations contributes tangible concepts for scrutiny.

While contemporary literature predominantly focuses on patient-centric perspectives, emphasizing specific architectural aspects through qualitative and quantitative studies, the professional-planning viewpoint remains sporadically explored, underscoring the innovative nature of this study. Aligned with the methodologies of Vollmer et al. [3] and Fricke et al. [5], addressing and probing the dissonance between users and planners is crucial for informed decision-making in extensive renovations and upcoming constructions. This study meticulously investigates and reveals four discrepancies, indicating a smaller dissonance than anticipated.

Within this investigation, it became evident that scientific insights in the field of Healing Architecture do not readily permeate the medical domain but often remain confined to architecture and engineering sciences. This poses a challenge as architecture, initially considered a non-medical intervention, demonstrates a measurable impact on healing and illness. This makes it pertinent and valuable for medical professionals, necessitating their awareness of Healing Architecture themes. Achieving this awareness requires brief, practical training sessions since these topics are not integrated into medical curricula or professional development guidelines, e. g. in Germany [26, 27]. The significance of such training becomes more pronounced when the aforementioned professionals are involved in planning and construction processes. On the other hand, it would be desirable for architects, who are aware of the positive effects of healing architecture, to be educated about the challenges and difficulties in practical implementation, thus integrating both perspectives.

The architecture of spaces where therapeutic interventions against the will of patients are implemented due to acute self-harm or harm to others (so-called coercive measure, e.g., restraints of seclusions) remains a largely unexplored field, especially in child and adolescent psychiatry. However, preliminary studies suggest that modern architecture tailored to the needs of psychiatric patients can reduce the incidence of coercive measures [28, 29]. The ARCHI questionnaire does not address this specific issue concerning the architecture of child and adolescent psychiatric facilities. Considering that coercive measures are recurrent in the acute care of child and adolescent psychiatry, incorporating this aspect into future research and potentially developing the ARCHI questionnaire accordingly could be beneficial.

In addition to the perspectives of senior physicians (representing the user group in this survey) and architects (representing the planners in this survey), it would be both valuable and intriguing to extend future surveys to the actual users. Given that children, adolescents, parents and caregivers are the target audience, conducting a survey among these individuals and comparing the results with the data generated in this study would be interesting.

## Relevance to child and adolescent mental health

Children and adolescents requiring inpatient psychiatric treatment spend significantly more time in child and adolescent psychiatric clinics than on any other (somatic) ward. These facilities predominantly embrace pedagogically structured, family-analogous, and system approaches, transforming the ward into a temporary home. This amplifies the relevance of architectural elements and potential influences. In the planning of new construction projects or renovations, medical personnel are often involved, albeit possessing limited expertise in successful and patient-oriented architectural concepts. Despite this, few disparities exist between architectural-planning and medical user perspectives. Primarily, the discrepancy arises from the inadequate transmission of new hospital architecture insights in medical education. Medical professionals engaged in planning and conceptual development for new or renovated projects are thus recommended to undergo training of further education in the realm of successful hospital architecture. This ensure that children and adolescents can optimally benefit from evolving scientific knowledge. It is crucial to recognise that architecture while a non-medical intervention, significantly impacts treatment outcomes.

## Strengths and limitations of the methodology, implementation and data analysis

This study presented firstly an innovative approach to address a carefully defined research gap, adhering to the traditional research paradigm of identifying a gap, employing high-quality methodology, objectively presenting results, and providing personal interpretation, discussion, and integration into the current research landscape. Utilizing the validated ARCHI questionnaire, the study aims to synergize insights and contribute to practical utility within the scientific community. Collaborating with architectural societies facilitated a comparatively large sample size, and despite modest participation, the study’s reach criterion is considered largely fulfilled, as it is comparable to sample sizes of similar surveys in the healthcare sector.

Acknowledging methodological limitations, socio-demographic data were not collected during the survey, impeding the analysis of the study population and adversely affecting the generalizability and external validity of the analysis. It’s crucial to note the survey’s focus on German child and adolescent psychiatric facilities, introducing potential selection and cultural biases, particularly in the demographics of the respondents. While the absence of qualitative data integration limits the depth of the analysis, the study’s hypotheses in the fourth chapter are explicitly presented as such, emphasizing the need for a qualitative examination of respondents.

## Data Availability

The data presented in this study are available on request from the corresponding author (DF). The data are not publicly available due to privacy reasons.

## Abbreviations

ARCHI: Questionnaire for evaluating the therapeutic relevance of architectural aspects in clinics or care facilities for child and adolescent psychiatry, psychosomatics, and psychotherapy for professionals working therapeutically within in the System

## References

[1] Frandsen AK. Helende Arkitektur. Arkitektur & Design: Aalborg Universitet; 2009.

[2] Lundin S. Healing Architecture: Evidence, Intuition, Dialogue. [Thesis for the degree of Licentiate of Architecture]: Chalmers University of Technology; 2015. Accessed December 30, 2023. https://publications.lib.chalmers.se/records/fulltext/223475/223475.pdf.

[3] Vollmer TC, Koppen G, Vraetz T, Niemeyer C. Entwicklungsräume JuKiP. 2017;06(06):239–44. 10.1055/s-0043-120229.

[4] Gong Y, Palmer S, Gallacher J, Marsden T, Fone D. A systematic review of the relationship between objective measurements of the urban environment and psychological distress. Environ Int. 2016;96:48–57. 10.1016/j.envint.2016.08.019. 27599349 10.1016/j.envint.2016.08.019

[5] Fricke OP, Halswick D, Längler A, Martin DD. Healing Architecture for Sick Kids. Z Kinder Jugendpsychiatr Psychother. 2019;47(1):27–33. 10.1024/1422-4917/a000635. 30560714 10.1024/1422-4917/a000635

[6] Becker K. Bedeutung der Architektur in der und für die Kinder- und Jugendpsychiatrie. Z Kinder Jugendpsychiatr Psychother. 2019;47(1):5–8. 10.1024/1422-4917/a000639. 30628864 10.1024/1422-4917/a000639

[7] Müller-Küppers M, Lehmkuhl U, Mahlke W. Die kinderpsychiatrische Klinik als Wohn- und Lebensraum - Erfahrungen bei der Neugestaltung einer kinder- und jugendpsychiatrischen Station. Prax Kinderpsychol Kinderpsychiatr. 1987;36(4):139–44. 10.23668/psycharchives.11484. 3615357

[8] Wöckel L, Rung D, Bachmann S, Dietschi H, Wild D. Burg Lino – Ein innenarchitektonisches Konzept zur Verbesserung der stationären Behandlung in der Kinder- und Jugendpsychiatrie. Z Kinder Jugendpsychiatr Psychother. 2019;47(1):19–26. 10.1024/1422-4917/a000641. 30558463 10.1024/1422-4917/a000641

[9] Dresler T, Rohe T, Weber M, Strittmatter T, Fallgatter AJ. Effects of improved hospital architecture on coercive measures. World Psychiatry. 2015;14(1):105–6. 10.1002/wps.20201. 25655168 10.1002/wps.20201 PMC4329907

[10] Evans GW. The built environment and mental health. J Urban Health. 2003;80(4):536–55. 10.1093/jurban/jtg063. 14709704 10.1093/jurban/jtg063 PMC3456225

[11] Beyer KMM, Kaltenbach A, Szabo A, Bogar S, Nieto FJ, Malecki KM. Exposure to neighborhood green space and mental health: evidence from the survey of the health of Wisconsin. Int J Environ Res Public Health. 2014;11(3):3453–72. 10.3390/ijerph110303453. 24662966 10.3390/ijerph110303453 PMC3987044

[12] Britton E, Kindermann G, Domegan C, Carlin C. Blue care: a systematic review of blue space interventions for health and wellbeing. Health Promot Int. 2020;35(1):50–69. 10.1093/heapro/day103. 30561661 10.1093/heapro/day103 PMC7245048

[13] Gascon M, Triguero-Mas M, Martínez D, et al. Mental health benefits of long-term exposure to residential green and blue spaces: a systematic review. Int J Environ Res Public Health. 2015;12(4):4354–79. 10.3390/ijerph120404354. 25913182 10.3390/ijerph120404354 PMC4410252

[14] McCormick R. Does access to green space impact the mental well-being of children: a systematic review. J Pediatr Nurs. 2017;37:3–7. 10.1016/j.pedn.2017.08.027. 28882650 10.1016/j.pedn.2017.08.027

[15] Jovanović N, Campbell J, Priebe S. How to design psychiatric facilities to foster positive social interaction - a systematic review. Eur Psychiatry. 2019;60:49–62. 10.1016/j.eurpsy.2019.04.005. 31112827 10.1016/j.eurpsy.2019.04.005

[16] Statistisches Bundesamt. Grunddaten der Krankenhäuser.: Statistischer Bericht - Grunddaten der Krankenhäuser 2022. Published December 4, 2023. https://www.destatis.de/DE/Themen/Gesellschaft-Umwelt/Gesundheit/Krankenhaeuser/Publikationen/_publikationen-innen-grunddaten-krankenhaus.html. Accessed 30 Dec 2023.

[17] Schepker R, Kölch M. Die Versorgungslandschaft der Kinder- und Jugendpsychiatrie und -psychotherapie in Deutschland: Strukturen. Herausforderungen und Entwicklungen Bundesgesundheitsblatt Gesundheitsforschung Gesundheitsschutz. 2023;66(7):745–51. 10.1007/s00103-023-03724-1. 37310425 10.1007/s00103-023-03724-1 PMC10328883

[18] Glasow N. Bauliche Suizidprävention in stationären psychiatrischen Einrichtungen. [Zugl.: Dresden, Techn. Univ., Fak. Architektur, Diss., 2011]. Logos-Verl; 2011.

[19] Scheiderer M-L, Reuter LM, Brockmann K, et al. ARCHI – Entwicklung eines Fragebogens zur Architektur kinder- und jugendpsychiatrischer Einrichtungen. Z Kinder Jugendpsychiatr Psychother. 2022;50(5):358–68. 10.1024/1422-4917/a000838. 34749537 10.1024/1422-4917/a000838

[20] Schmidt P, Reis D, Schulte AG, Fricke O. Self-assessment of knowledge on the treatment of children and adolescents with special care needs: results of a survey amongst German dentists with key expertise in paediatric dentistry. J Pers Med. 2022;12(7). 10.3390/jpm12071173. 10.3390/jpm12071173 PMC9319936 35887670

[21] Fornefeld D, Fricke O, Schulte AG, Schmidt P. Investigation of dental and oral health in children and adolescents with special support needs from a child and adolescent psychiatric perspective. Children (Basel). 2024;11(3):355. 10.3390/children11030355. 38539390 10.3390/children11030355 PMC10969292

[22] Gibbons JS, Butler JP. Quality of life for “new” long-stay psychiatric in-patients. The effects of moving to a hostel. Br J Psychiatry. 1987;151:347–54. 10.1192/bjp.151.3.347. 3122867 10.1192/bjp.151.3.347

[23] Wood VJ, Curtis SE, Gesler W, et al. Creating “therapeutic landscapes” for mental health carers in inpatient settings: a dynamic perspective on permeability and inclusivity. Soc Sci Med. 2013;91:122–9. 10.1016/j.socscimed.2012.09.045. 23261254 10.1016/j.socscimed.2012.09.045

[24] Whitehead CC, Polsky RH, Crookshank C, Fik E. Objective and subjective evaluation of psychiatric ward redesign. Am J Psychiatry. 1984;141(5):639–44. 10.1176/ajp.141.5.639. 6711683 10.1176/ajp.141.5.639

[25] Lee E, Daugherty J, Selga J, Schmidt U. Enhancing patients’ wayfinding and visitation experience improves quality of care. J Perianesth Nurs. 2020;35(3):250–4. 10.1016/j.jopan.2019.11.003. 32113796 10.1016/j.jopan.2019.11.003

[26] Ärztekammer Westfalen-Lippe. (2022). Weiterbildungsordnung der Ärztekammer Westfalen-Lippe vom 02. April 2022 inkl. Richtlinien über den Inhalt der Weiterbildung. https://www.aekwl.de/fileadmin/user_upload/aekwl/weiterbildung/Aenderungsfassung_April_2022_-_WO_AEKWL_01.07.2023.pdf. Accessed 30 Dec 2023.

[27] Bundesärztekammer. (2020). (Muster-)Fachlich empfohlene Weiterbildungspläne. Fachlich empfohlener Weiterbildungsplan für den/die Facharzt für Kinder- und Jugendpsychiatrie und -psychotherapie. https://www.bundesaerztekammer.de/fileadmin/user_upload/BAEK/Themen/Aus-Fort-Weiterbildung/Weiterbildung/FEWP/FA_SP-WB/20201112_13_FEWP_KJPP.pdf. Accessed 30 Dec 2023.

[28] Czernin K, Bründlmayer A, Baumgartner JS, Plener PL. Space for youth mental health – coercive measure use before and after architectural innovation at a department of child and adolescent psychiatry. Child Adolesc Ment Health. 2023. 10.1111/camh.12690. 38084775 10.1111/camh.12690

[29] Rohe T, Dresler T, Stuhlinger M, Weber M, Strittmatter T, Fallgatter AJ. Bauliche Modernisierung in psychiatrischen Kliniken beeinflussen Zwangsmaßnahmen. Nervenarzt. 2017;88(1):70–7. 10.1007/s00115-015-0054-0. 26820456 10.1007/s00115-015-0054-0

